# Maternal expectations of fetal gender and risk of postpartum depression

**DOI:** 10.1186/s12884-023-05419-6

**Published:** 2023-02-13

**Authors:** Xiao-Fan Rong, Ming-Qing Li, Faustino R. Pérez-López, Jiang-Nan Wu, Bin Zhang

**Affiliations:** 1 Obstetrics and Gynecology Hospital, Fudan University, Shanghai, 200001 China; 2 Qingpu Branch of Zhongshan Hospital, Fudan University, Shanghai, 201700 China; 3 Shanghai Key Laboratory of Female Reproductive Endocrine-Related Diseases, Shanghai, 200011 China; 4 Aragón Health Research Institute, San Juan Bosco 13, 50009 Zaragoza, Spain; 5 Department of Obstetrics and Gynecology, University of Zaragoza Faculty of Medicine, Domingo Miral s/n, 50009 Zaragoza, Spain

**Keywords:** Postpartum depression, Gender expectations, Cohort study, Primiparous women, Pluriparous women

## Abstract

**Background:**

Female offspring was associated with a high risk of postpartum depression (PPD) during the one-child policy period in China. However, little is known about the association between maternal expectations on fetal gender and the risk of PPD in the context of the new two children policy implemented in 2016.

**Methods:**

We conducted a hospital-based cohort study of women with singleton pregnancies between 2017 and 2018 (*n* = 991) to address this concern. Logistic regression was run to estimate the association between unexpected fetal gender and the risk of PPD.

**Results:**

A total of 127 women (12.8%) were diagnosed with PPD. Compared with women who achieved fetal gender expectations, the odds ratio (OR) for PPD among those who had an unexpected fetal gender was 2.44 (95% confidence interval (CI): 1.30–4.58) (in the backward method logistic regression model) and 2.25 (95% CI: 1.21–4.18) (in the forward method model), respectively. The disparity of the association was significant among primiparous and pluriparous women (OR, 2.52, 95% CI: 1.32–4.84, *P *= 0.005 vs. OR, 0.91, 95% CI: 0.09–8.75, *P* = 0.932). Fetal gender expectations accounted for about 15% of the risk of PPD in the structural equation models.

**Conclusions:**

These results indicated that unexpected fetal gender was associated with an increased risk of PPD among Chinese primiparous women.

## Background

Postpartum depression (PPD) affects 5.0% to 56.1% of pregnant women worldwide [1, 2]. The disorder is characterized by persistent low emotion in the puerperium and may impact the mother's quality of life and family functioning, resulting in consequences for offspring development and mental health [3, 4]. However, PPD is generally considered a multifactorial disease [5, 6]. Abuse in childhood or by an intimate partner, maternal low educational attainment, low socioeconomic status at the time of pregnancy, lack of social support, and a history of mental illness have been identified as risk factors for PPD [1, 5]. In addition, although the association between fetal gender and the risk of PPD remains controversial in observational studies [7, 8], the majority of evidence supports the conclusion that a daughter was associated with an increased risk of PPD [9–12].

Although a female child might be a preference for some people, male fetuses are generally preferred to female fetuses [13]. The prejudice might cause sex-selective abortion and result in gender imbalance in China. Because men are the main labor force, the concept of "son preference" has existed in China for more than 2000 years. Many efforts, including publicity of gender equality and prohibiting non-medical identification of fetal gender, have been implemented to eliminate the son preference of parents in China. However, son preference is still deeply rooted in the rural areas with backward economy. According to the sixth nationwide population census, the sex ratio was 104.9 males for every 100 females in 2011 [14]. China has changed its restrictions on the fertility policy since 2016, allowing each family to have two children [15]. The new policy may adjust the traditional son preference since each family can avail of the opportunity to have two children, instead of the “one family one child” policy before 2016. Thus, fetal gender expectations, rather than fetal gender itself, may reflect a maternal real attitude towards offspring reproduction. Furthermore, fetal gender identification for nonmedical reasons is illegal and forbidden during pregnancy in China. The sudden unexpected fetal gender after birth might have a further impact on the postpartum maternal psychological state. We assumed that fetal gender expectations rather than fetal gender itself may be better in evaluating the risk of PPD. We conducted a retrospective study to address this concern and to quantify the extent of the impact of gender expectations on the risk of PPD.

## Methods

### Study design

We conducted a hospital-based retrospective study of women who have their postpartum clinical exams on the sixth week after birth at the Obstetrics and Gynecology Hospital of Fudan University (Shanghai, China), between January 2017 and January 2018. The Chinese version of the Edinburgh Postpartum Depression Scale (EPDS) questionnaire has been previously validated in our pilot study and previous studies conducted in other regions in China [16, 17]. We used this validated questionnaire to assess maternal mental status.

Maternal basic characteristics (e.g., maternal age at birth, parity, and education level) were surveyed. In addition, postpartum maternal and family information was also recorded, including family economic and relationship satisfaction, fetal gender expectations, infantile feeding mode, infantile growth status, and husband’s help in baby care. Pregnancy outcomes, including gestational age, birth mode, infant’s gender, and birth weight, were extracted from the electronic medical records.

PPD usually occurs within six weeks postpartum, continuing after the puerperium, as far as individual women continue the infant care until the child goes to school [18]. Women delivered in our hospital are required to attend an outpatient follow-up consultation around the sixth week after birth, which allows for obtaining complementary information. The study protocol was approved by the Ethics Committee of the Obstetrics and Gynecology Hospital of Fudan University. Written informed consent was obtained from all women before the investigation. The authors obtained administrative permission to collect the data for research purposes.

### Exposure and outcomes

Fetal gender expectations were surveyed at the postpartum examination conducted in the sixth week after birth. Exposure was defined as the discrepancy between actual fetus’ gender and maternal expectations. Various cut-off values of the EPDS for diagnosing PPD may result in different screening values. In a recent meta-analysis, an EPDS cut-off value of 11 or higher maximized combined sensitivity and specificity (sensitivity = 0.81, specificity = 0.88, Youden’s J = 0.69), while a cut-off value of 13 or higher was less sensitive but more specific (sensitivity = 0.66, specificity = 0.95, Youden’s J = 0.61) [19]. However, a cut-off value of 9 or higher had a satisfactory value in screening PPD in Chinese women, with a sensitivity and specificity of 0.82 and 0.86, respectively [20]. Therefore, a cut-off value of 9 or higher was referred as the criteria for PPD in the present study. The Cronbach’s alpha of EPDS was 0.769.

### Statistical analysis

Categorical data were expressed as n (%) between women with and without PPD. Differences between the two groups were compared by chi-square or Fisher’s exact test. Logistic regression models, including a conditional backward or forward method; were run to screen factors significantly associated with the risk of PPD. All variables were involved in the screening progression. The exclusion and inclusion criteria p-value for variables selection in the models were 0.10 (for the backward method) and 0.05 (for the forward method), respectively. Odds ratios (ORs) and the corresponding 95% confidence intervals (CIs) for PPD were estimated for women who had an offspring of unexpected gender, compared with those who had an offspring’s gender consistent with the fetal gender expectation. Since parity and son preference might affect the fetal gender expectation and PPD association, stratification analysis according to parity (1 or > 1) and fetal gender (male or female) was further performed to examine the differences of the ORs between the subgroups. Maternal education level (junior or below, senior, university, or master degree and above), family economic satisfaction (yes or no), and family relationship satisfaction (yes or no) were also included in the adjusted models.

Structural equation models (SEMs) were constructed to study the effect of variables on the risk of PPD and to quantify the effect size of unexpected fetal gender on the risk of PPD. The variables that were screened by the forward method (four variables: gender expectation, family relationship satisfaction, maternal education level, and economic dissatisfaction) were included in the SEM1, while an additional variable (the four variables in the SEM1 plus infantile growth) was further included in the SEM2.

All statistical tests were conducted using IBM SPSS Statistics version 22.0 (including Amos version 20). A two-sided *P* value < 0.05 was considered statistically significant.

## Results

### Base characteristics

**Fig. 1:**
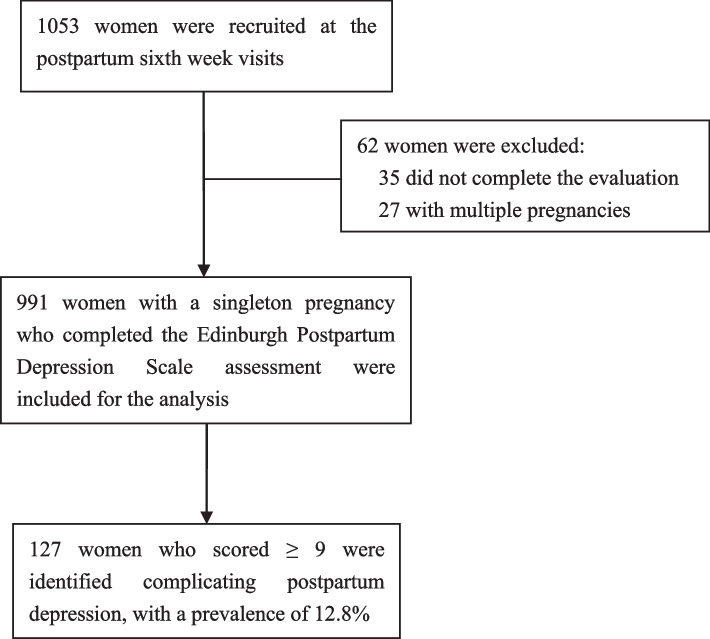
Flow chart of the study

A total of 1053 women were registered at their postpartum six week visit, after excluding 35 women who did not complete the EPDS evaluation and 27 women with multiple pregnancies, 991 women (94.1%) were included in the present study. Among these women, 127 women (12.8%) scored ≥ 9 in the EPDS evaluation and were diagnosed with PPD (Fig. 1). Maternal age and education level gained help from the husband during baby care, satisfaction with economic and family relationships, and fetal gender expectations were unbalanced between women with and without PPD, while no significant differences were found for the other variables (Table 1).

**Table 1:** Baseline characteristics between women with and without postpartum depression

Variables	PPD				Chi-square value	*P* value
	No(*N* = 864)		Yes(*N* = 127)			
Gender satisfaction	811	93.9%	110	86.6%	8.87	0.003
Maternal age (years)					7.10	0.029
25–34	750	86.8%	99	78.0%		
< 24	35	4.1%	9	7.0%		
≥ 35	79	9.1%	19	15.0%		
Maternal education level					35.51	< 0.001
Junior or below	34	3.9%	18	14.2%		
Senior	71	8.2%	21	16.5%		
University	636	73.6%	77	60.6%		
Master degree or above	123	14.2%	11	8.7%		
Economic satisfaction	828	95.8%	107	84.3%	27.856	< 0.001
Family relationship satisfaction	848	98.1%	110	86.6%	45.758	< 0.001
Preterm	47	5.4%	10	7.9%	1.21	0.271
Parity = 1	777	89.9%	114	89.8%	0.01	0.954
Birth mode					2.05	0.358
Natural	495	57.3%	77	60.6%		
Cesarean	334	38.7%	48	37.8%		
Forceps	35	4.1%	2	1.6%		
Male infants	449	52.0%	71	55.9%	0.688	0.407
Birth weight (g)					0.618	0.734
< 2500	33	3.8%	5	3.9%		
2500–4000	759	87.8%	114	89.8%		
> 4000	72	8.3%	8	6.3%		
Feeding mode					0.384	0.825
Breast milk	367	42.5%	51	40.2%		
Powdered milk	87	10.1%	12	9.4%		
Mixed	410	47.5%	64	50.4%		
Growth status					2.91	0.233
Good	809	93.6%	120	94.5%		
Fair	54	6.3%	6	4.7%		
Poor	1	.1%	1	.8%		
Gain help from husband	831	96.2%	117	92.1%	4.385	0.036

### Factors associated with the risk of PPD

The logistic regression model by a forward (conditional) method indicated that maternal education level, satisfaction with economic and family relationships, and fetal gender expectations were included in the model (Table 2). In the model by a backward (conditional) method, maternal education level, satisfaction with economic and family relationships, infantile growth, and fetal gender expectations remained in the model, while other variables were excluded (Table 2). Higher maternal education levels were associated with a lower risk of PPD, while dissatisfaction with family economics and relationships, and unexpected fetal gender were risk factors for PPD. Compared with mothers who achieved fetal gender expectations, the OR for PPD among those who had unexpected fetal gender offspring was 2.44 (1.30–4.58) (in the backward method) and 2.25 (1.21–4.18) (in the forward method), respectively (Table 2).

**Table 2:** Factors associated with the risk of postpartum depression

Variables	Forward method				Backward method			
	OR	lower limit	upper limit	P value	OR	lower limit	upper limit	*P* value
Unexpected gender	2.249	1.211	4.175	0.010	2.441	1.302	4.575	0.005
Maternal education level								
Junior	1.000				1.000			
Senior	0.549	0.252	1.193	0.130	0.522	0.239	1.143	0.104
University	0.219	0.116	0.416	< 0.001	0.202	0.106	0.385	< 0.001
Master degree or above	0.181	0.076	0.430	< 0.001	0.162	0.067	0.387	< 0.001
Economic dissatisfaction	2.760	1.440	5.290	0.002	2.907	1.494	5.657	0.002
Family relationship dissatisfaction	6.545	3.043	14.076	< 0.001	7.049	3.266	15.215	< 0.001
Growth status								
Good					1.000			
Fair					0.368	0.141	0.961	0.041
Poor					4.421	0.170	115.202	0.372

Unexpected fetal gender remained significantly associated with the risk of PPD among primiparous women (OR 2.52, 95% CI: 1.32–4.84, *P* = 0.005), while no significant association was found for pluriparous women (Table 3). However, among primiparous women, the relationship between unexpected fetal gender and the risk of PPD in women who give birth to male fetuses differed from those who have a female fetus (P values for the ORs were 0.031 and 0.078, respectively; Table 3).

**Table 3:** Association between unexpected fetal gender and the risk of postpartum depression, by parity and fetal gender^*^

Actual fetal gender	Expected fetal gender	Parity = 1 (*N* = 891)		Parity > 1 (*N* = 100)	
		OR (95% CI)	*P* value	OR (95% CI)	*P* value
Male	Female	2.44 (1.09–5.50)	0.031	0.95 (0.08–10.23)	0.945
Female	Male	2.68 (0.90–8.00)	0.078	-	
Total	Total	2.52 (1.32–4.84)	0.005	0.91 (0.09–8.75)	0.932

^*^ Control for maternal education level, family economic, and relationship satisfaction

### Extent of the effect of fetal gender expectation on PPD

**Fig. 2:**
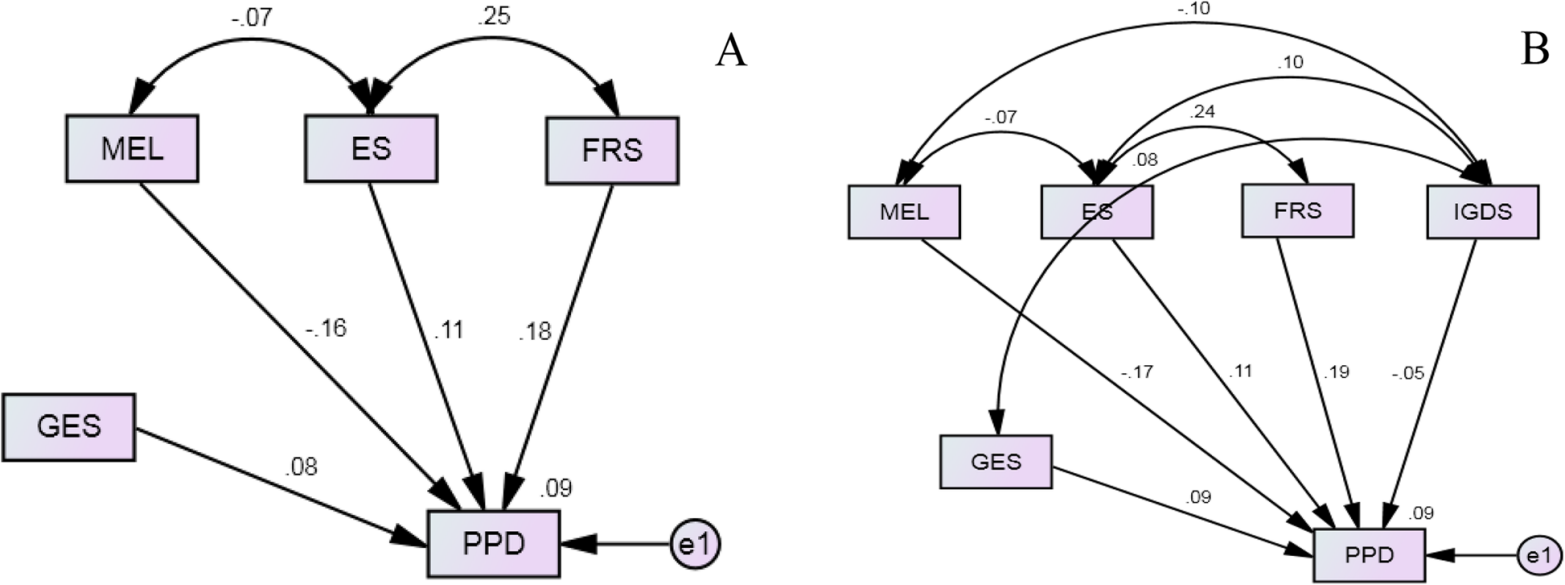
Structural equation model for the risk of PPD. **A**: GES worked independently and directly, while the other three factors (MEL, ES, and FRS) were correlated and worked directly with the development of PPD in SEM1. **B**: All five factors were correlated (GES was only correlated with IGDS, and the other four factors were correlated with each other) and worked directly on the occurrence of PPD in SEM2. MEL: Maternal Education Level; ES: Economic Satisfaction; GES: Gender Expectation Satisfaction; FRS: Family Relationship Satisfaction; IGDS: Infantile Growth and Development Status; PPD: Postpartum Depression;e 1: Error variable 1

Two SEMs were constructed to quantify the effect sizes of factors selected from the two logistic regression models (i.e., the forward and backward method, respectively) on the risk of PPD. The SEM1 that contained maternal education level, satisfaction with economic and family relationships, and fetal gender expectation satisfaction fitted well (Chi-square = 0.289, df = 4, *P* = 0.576, RMSEA = 0.000, AGFI = 0.996) and may explain 9.1% of individual variations of the PPD risk (Fig. 2 A). Standardized estimates of the four factors ranged from -0.161 to 0.185 and all significantly differed from 0 (all *p* < 0.01), the effect extent of fetal gender expectation achievement accounted for 15.30% of the total risk of PPD (Table 4). Similar results were found in the SEM2 in which an additional variable (infantile growth status) was included (Fig. 2 B, Table 4).

**Table 4:** Effect and extent of the impact factors on the risk of postpartum depression

Variables	SEM1		SEM2	
	Standardized effect estimates	Size of effect (%)	Standardized effect estimates	Size of effect (%)
Gender expectation	0.082^*^	15.30	0.086^*^	14.43
Family relationship satisfaction	0.185^**^	34.51	0.185^**^	31.04
Maternal education level	-0.161^**^	30.04	-0.165^**^	27.68
Economic dissatisfaction	0.108^**^	20.15	0.113^**^	18.96
Infantile growth	-	-	-0.047	7.89

^*^P < 0.01, ^**^P < 0.001

## Discussion

In this retrospective cohort study of 991 women with single pregnancies, we found that maternal–fetal gender expectation rather than fetal gender itself was associated with and accounted for about 15% of the risk of PPD. Maternal education level and subjective feelings of family economy and relationships were also correlated with the occurrence of PPD. Among these factors, family relationship dissatisfaction is the most important factor for PPD occurrence, accounting for over 31% of the PPD risk in the SEM models in the present study.

Consistent with previous studies, we found that maternal dissatisfaction with family economics and relationships, low education attainment were linked to an increased risk of PPD, which may be attributed to the initiation of income-related stressors (e.g., financial strain), a lack of social support, and low health literacy and the inability to recognize symptoms of depression and to seek help [1, 21, 22].

In previous studies, maternal low educational attainment, low socioeconomic status, lack of social support, and history of domestic violence and mental illness have been consistently associated with the risk of PPD. However, the association between fetal gender and the risk of PPD is still controversial. Female infants are associated with a higher risk of PPD in some Asian countries with special cultures, such as India [1, 23]. In China, this association varied according to the time of the study. Positive correlations were found in studies conducted around 2010 [12, 24], while weakened or null associations have been identified in recent studies since 2015 [25–27]. Our findings are consistent with the latest studies. Compared with women having a daughter, the adjusted OR for PPD compared to those with a son was 0.87 with a 95% CI of 0.58–1.31 (*P* = 0.51).

Interestingly, unexpected fetal gender was associated with an increased risk of PPD. The result seemed inconsistent with son preference, which was expected to show a bigger effect compared with daughter preference with unexpected fetal gender. A possible explanation is that the new “one family two children” policy implemented in 2016 has alleviated the maternal stress of son preference. In the context of having an added opportunity for more than one pregnancy/child, a daughter might also be acceptable for pregnant women. This point of view may be further supported by the stratification findings. In the stratification analyses, unexpected fetal gender was associated with an increased risk of PPD in primiparous women but not for pluriparous women (P values for adjusted OR were 0.005 and 0.932, respectively). In addition, among the primiparous women, this association remained for women who expected a daughter, while a null association was found for those expecting a son (P values were 0.031 and 0.078, respectively). The difference might reflect the releasing of diversified gender demands of mothers in the new policy.

In the context of the relaxation of the new two children policy, unexpected fetal gender means differently between primiparous and pluriparous women. Pluriparous women might have higher acceptance of the result and clearer expectations for the future (*e.g.*, they do not need to be pregnant again). However, unexpected fetal gender might bring greater emotional and economic pressure to primiparous women. For them, an unexpected fetal gender possibly implies a new pregnancy [22]. A new pregnancy means more maternity leave, work interruptions, and readaptation of work, which might lower the mother’s professional evaluation from the leader and colleagues and then result in an increased risk of physiological and psychological pressure. Son preference is a common concept in the context of only one child policy, 30% of survey respondents intended to have a son while only 16% hoped to have a daughter [13]. However, family economic levels might affect the maternal expectations of fetal gender since a son always means to bear the high economic pressure formed by marriage, such as buying a new house for marriage and a large amount of dowry. Primiparous women who expect a daughter possibly already face a great economic pressure, giving birth to a son will further put more financial and psychological pressure on the family. According to the seventh population census, the male to female ratio is 105.1 for 100 in 2021. Women who prefer a son have a high socioeconomic status, put more emphasis on the identity of partners, and have a stronger earning power in the process of marriage. This means that men need to have some financial ability to get married. In China's family-oriented society, men's marriage problem is never a personal, but a family problem. Therefore, the existence of older unmarried men is undoubtedly a pressure on every member of the family. According to China's customs, men should provide new houses and betrothal gifts (the man needs to give the woman's parents a sum of money before marriage). In recent years, the overall house price is still in a very high level, and the rise of house prices is still around 7% [28]. In many places, betrothal gifts are often hundreds of thousands, which increases the economic pressure on male baby mothers. Traditional son preference holders think that men's labor ability and economic benefits are significantly higher than women's, and men's social competitiveness and economic advantages are higher than women's. They hold the view that a son is a better option than a daughter. Primiparous women who expect a son are less likely to have a second child [13]. They might plan a new pregnancy when the fetal gender is inconsistent with the expectation. The increased risk of PPD of unexpected fetal gender might be attributed to the disappointment of fetal gender and the fear of an additional dissatisfaction with fetal gender in a new pregnancy in the context of the new two children policy.

The findings in the present study have a great clinical value for possible interventions for pregnant women. Screening and treatment for depression may reduce depressive symptoms and then decline the prevalence of PPD [2, 4]. We firstly found a new PPD susceptible group of pregnant women who hold a different view from the traditional son preference concept. We further found that, in the SEM, the impact of fetal gender expectations on the risk of PPD was direct. Its impact was not correlated with other socioeconomic factors, such as family economic status and maternal educational level. Therefore, for women at high risk of PPD, clinicians and family members may carry out appropriate publicity, education, and interventions (rather than gender equality) to downplay the harms of fetal gender expectations. Moreover, the regulations of fetal gender identification might be adjusted, such as allowing pregnant women to know the fetal gender during pregnancy. Earlier fetal gender identification might enhance the acceptance of the fetuses of the mother and cushion the impact of sudden unexpected infant gender on subsequent maternal mental health. Furthermore, because that maternal education level and family economic status are hard to change in a short term, harmonious family relationship constructions during pregnancy and/or in lactation are then suggested to reduce the attributed risk of PPD.

Our study has some limitations. First, the questionnaire was designed relatively simply to improve the compliance of participants. For example, only two grades instead of hierarchical options were available for some items. We included a few factors that are associated with the risk of PPD. These shortages might result in insufficient factors for the comprehensive analysis in the SEM, in which only about 9.1% of individual variations of the PPD risk could be explainable.

Second, this was a single-center study and we only evaluated less than 10% of the annual deliveries at the hospital. Furthermore, women delivered at the hospital were thought to have higher education level and income level [29, 30]. Therefore, the representativeness of the sample might be limited, as well as the generalizability of these findings. Third, a score of ≥ 9 might be a sensitive but not a common measure of PPD (e.g., ≥ 10 or ≥ 13). In addition, evidence shows that there is a difference in the severity of symptomatology between women with EPDS 10–12 and those with EPDS > 12 [19]. Consequently, there may be a potential overestimation of the prevalence of PPD and an overestimation of the independent variable effect on PPD. Finally, we determined the exposure and PPD at the same time. Although gender expectations are generally consistent throughout pregnancy, we could not exclude the potential impact of PPD on participants’ recollection of fetal gender expectations. Thus, the interpretation of causal relationships should be cautious.

In conclusion, maternal expectation of fetal gender was a special indicator and was associated with the risk of PPD. In the new context of two-child policy, measures are suggested to weaken the maternal–fetal gender expectation and then reduce the risk of PPD, especially for primiparous women.

## Data Availability

The datasets used in the present study are available from the corresponding author on reasonable request.

## Abbreviations

CI: Confidence interval
EPDS: Edinburgh postpartum depression Scale
OR: Odds ratio
PPD: Postpartum depression
SEMs: Structural equation models
